# Inconsistency of in vitro exsheathment triggers for gastrointestinal nematode parasites of sheep, cattle and deer

**DOI:** 10.1007/s00436-024-08277-z

**Published:** 2024-07-11

**Authors:** Kiliana Bekelaar, Luis Carvalho, Tania Waghorn, Peter Green, Charlotte Bouchet, Dave Leathwick

**Affiliations:** Grasslands Research Centre, AgResearch Ltd, Private Bag 11008, Palmerston North, New Zealand

**Keywords:** Exsheathment, Temperature, pH, Carbon dioxide, Nematode, Strongyles

## Abstract

Exsheathment is crucial in the transition from free-living to parasitic phase for most strongyle nematode species. A greater understanding of this process could help in developing new parasitic control methods. This study aimed to identify commonalities in response to exsheathment triggers (heat acclimation, CO_2_ and pH) in a wide range of species (*Haemonchus contortus*, *Trichostrongylus* spp.,* Cooperia* spp., *Oesophagostomum* spp.,* Chabertia ovina*, and members of the subfamily Ostertagiinae) from sheep, cattle and farmed deer. The initial expectation of similarity in pH requirements amongst species residing within the same organ was not supported, with unexpected pH preferences for exsheathment of *Trichostrongylus axei*, *Trichostrongylus vitrinus*, *Trichostrongylus colubriformis* and *Cooperia oncophora.* We also found differences between species in their response to temperature acclimation, with higher exsheathment in response to heat shock observed for *H. contortus*, *Ostertagia ostertagi*, *T. axei*, *T. vitrinus* and *Oesophagostomum sikae*. Furthermore, some species showed poor exsheathment under all experimental conditions, such as *Cooperia curticei* and the large intestinal nematodes *C. ovina* and *Oesophagostomum venulosum*. Interestingly, there were some significant differences in response depending on the host from which the parasites were derived. The host species significantly impacted on the exsheathment response for *H. contortus*, *Teladorsagia circumcincta, T. vitrinus* and *T. colubriformis*. Overall, the data showed variability between nematode species in their response to these in vitro exsheathment triggers, highlighting the complexity of finding a common set of conditions for all species in order to develop a control method based on triggering the exsheathment process prematurely.

**Supplementary Information:**

The online version contains supplementary material available at 10.1007/s00436-024-08277-z.

## Introduction

Parasitic infections in grazing ruminants are a major issue for farmers due to their potential impact on both the animal’s health and the farm’s economy (Emery et al. 2016; Mavrot et al. 2015; Roeber et al. 2013). For decades, anthelmintics have been the cornerstone of parasite control, but today, resistance amongst nematode populations to these drugs is challenging the sustainability of many farming operations (Traversa & von Samson-Himmelstjerna 2016; Lamb et al. 2017; Baudinette et al. 2022; Sauermann et al. 2024). In the absence of new classes of anthelmintics, alternative approaches to parasite control, capable of reducing or eliminating the reliance on anthelmintics, will be required. To meet this need, a better understanding of the fundamental biology of helminths offers the potential identification of novel targets (vulnerabilities) for new interventions.

A critical process in the life cycle of most strongyle nematode species is the transition from the free-living to the parasitic phases. The free-living third stage larva (L3) is fully encapsulated by the retained sheath of the second stage, protecting it from environmental factors while on pasture (Rogers and Sommerville 1963; Sutherland and Scott 2010). In most species, after ingestion by a suitable host, this second stage sheath is shed (i.e. exsheathment) and development into an adult helminth is initiated (Sutherland and Scott 2010). Inhibition of exsheathment inside the host, or stimulation of this process on pasture prior to ingestion, would provide an attractive pathway for disruption of the life cycle and therefore parasite control. A thorough understanding of the stimuli triggering exsheathment across a range of parasite and host species is required as a foundation for such an approach.

A combination of heat shock and carbon dioxide (CO_2_), when applied at the appropriate pH, will trigger exsheathment in some, but not all, nematode species in vitro (Bekelaar et al. 2018a, b,^ 2019^; Rogers 1960; Rogers and Sommerville 1960; Taylor and Whitlock 1960). A previous study (Bekelaar et al. 2019) suggested that, for some abomasal species, factors additional to heat shock and CO_2_ were necessary for in vitro exsheathment, implying some variation between species in their exsheathment triggers. Appropriate pH is a requirement for optimal exsheathment and this is expected to vary depending on the organ in which each species would normally exsheath (Hertzberg et al. 2002; Rogers 1960; Silverman and Podger 1964). For example, *Haemonchus contortus*, which resides in the abomasum, is expected to exsheath in the neutral environment of the rumen whereas *Trichostrongylus colubriformis*, which resides in the small intestine, is expected to exsheath in the acid environment of the abomasum (Sommerville 1954). The current study aimed to expand on previous work and investigate the requirements for exsheathment in a wider range of abomasal and intestinal nematode species, to look for commonality in their triggers, as well as any differences associated with geographic location and host species.

## Methods

### Collection of faeces

Sheep, cattle or deer faeces were collected from farms across New Zealand. As the trial made use of convenient sampling opportunities, stock class and sampling times varied across the farms. Sheep (SF), cattle (CF) and deer (DF) farms (farm location, stock class, collection date) included in the trial were as follows: SF1 (Southland, lambs and mixed age ewes, July 2017), SF2 (Southland, lambs, March 2018), SF3 (Ruapehu, lambs, January 2018), SF4 (Rangitikei, lambs and mixed age ewes, October 2018), CF1 (Waitaki, calves, May 2018), CF2 (Taupo, heifers, July 2017), CF3 (Manawatu, calves, April 2018), CF4 (Waikato, adults, May 2021), CF5 (Rotorua, adults, May 2021), DF1 (Central Hawke’s Bay, weaners, July 2017), DF2 (Dunedin, adult deer, February 2018), DF3 (Manawatu, yearlings, July 2018).

Fresh faeces from multiple animals were collected from pasture after direct observation of defecation, and the bulk sample transported anaerobically to the AgResearch parasitology laboratory (Palmerston North, New Zealand) as described by Waghorn et al. (2018).

### Larval cultures

The faeces from each farm were mixed with vermiculite and cultured for approximately 14 days at 23 °C before L3 extraction using the Baermann technique (Hendrix 1998). Larvae were then stored at 10 °C and cleaned by migration through a 20-µm nylon mesh prior to use, to ensure larval viability and motility.

### In vitro exsheathment assay

Larvae were exposed to three exsheathment triggers simultaneously: (i) high levels of CO_2_, (ii) a pH which was either low (pH 2) or neutral (pH 6.6) and (iii) one of three temperature regimes, i.e. a heat shock (a rapid change from 20 to 40 °C and maintained at 40 °C), a slow temperature increase (slow change from 20 to 40 °C over 4 h and maintained at 40 °C) or a constant 20 °C. Three subsets of untreated L3 were killed using Lugol’s iodine solution just prior to the start of the experiment and used as a control. This control was used to verify that no exsheathment was present at the start of the experiment, and to control that the treatments do not change the species composition of the samples. Three replicates were included for each of the seven treatment conditions (two pH levels × three temperature treatments plus untreated control), resulting in 21 per farm.

The exsheathment assay was conducted within 6 months of sample collection as described by Bekelaar et al. (2018b) with minor modifications. Briefly, cleaned L3 (*n* = approximately 600) in 1 mL of tap water at room temperature were placed in polypropylene tubes and 9 mL of CO_2_-saturated buffer with a final pH of 6.6 or 2 was added to each tube. For samples exposed to heat shock the buffer was preheated to 40 °C, whereas for the other temperature regimes the buffer was at 20 °C when added to the larval suspension.

The buffer used was a 10 mM phosphate buffer (3.75 mM Na_2_HPO_4_, 6.25 mM KH_2_PO_4_, 0.5 mM MgCl_2_ and 0.5 mM CaCl_2_). At pH 6.6 the buffer was supplemented with NaHCO_3_ to a final concentration of 72.5 mM, which had previously been demonstrated to provide an optimal environment to achieve CO_2_-saturation (Bekelaar et al. 2018b). For experiments at pH 2, NaHCO_3_ was not added, and the pH was lowered by dropwise addition of 6 M HCl. For both solutions, CO_2_-saturation was obtained by bubbling with 100% CO_2_ for 1 h prior to use.

Larvae were killed 24 h after exposure to the exsheathment triggers by addition of 1 drop of 3% Lugol’s iodine solution to each tube. Tube contents were thoroughly mixed before 1.5 mL of the larval suspension was transferred to a flat bottom watch glass and L3 were subsequently examined at × 57 magnification using a stereomicroscope (Olympus SZX9). Exsheathment was defined as a complete or partial loss (at least 30% of the body had emerged) of the sheath. For the treated samples where exsheathed L3 were present and for the untreated controls, species identification was performed on all L3 sampled from the three replicates. For treatment conditions where no exsheathment was observed in any of the 1.5 mL subsamples for the three replicates, species composition was verified using a small aliquot from one replicate.

### Species identification

Each individual larva present in the 1.5 mL subsample had its exsheathment status recorded, and was then placed in a 200 µL thin-wall strip tube containing 10 µL of lysis solution (2.5% Proteinase K (Roche, Basel, Switzerland) in DirectPCR Lysis Reagent (Tail) (Viagen Biotech Inc, Los Angeles, CA, USA). Larvae were lysed for 16 h at 55 °C and 1 h at 90 °C and cooled to 12 °C. Lysates were diluted 1 in 3 with sterile water, thoroughly vortexed and kept at − 20 °C before being used in species identification assays.

Species identification was performed using multiplex species-specific PCRs targeting the second internal transcribed spacer (ITS-2) region of ribosomal DNA (Bisset et al. 2014; Tapia-Escarate et al. 2020). Five sets of reactions using multiple species-specific primers (Table 1) were run to target *Trichostrongylus vitrinus*, *Trichostrongylus axei*, *T. colubriformis*, *Teladorsagia (Ostertagia) circumcincta* and *Chabertia ovina* (Reaction 1); *Cooperia curticei*, *Cooperia oncophora, Nematodirus spathiger*, *H. contortus* and *Oesophagostomum venulosum* (Reaction 2); *Cooperia punctata*, *Ostertagia ostertagi*, *Ostertagia leptospicularis* and *Nematodirus filicollis* (Reaction 3); *Spiculopteragia asymmetrica*, *Spiculopteragia spiculoptera*, *O. venulosum*, *Oesophagostomum radiatum* and *Oesophagostomum sikae* (Reaction 4); and *Trichostrongylus askivali* (Reaction 5). Cross-reactions occurring with some of the primers (Table 1) were handled as described by Tapia-Escarate et al. (2020). PCR products were analysed by 2% agarose gel electrophoresis.

**Table 1:** List of primers and PCR conditions. Cross-reactions of primers with other species indicated with ^*^,^°^,^§^,^#^

Target	Primer	Assay	Sequence (5′-3′)	Length	PCR conditions
ITS2 generic	**F**	**all**	CACGAATTGCAGACGCTTAG	371–394	
ITS2 generic	**R**	**all**	GCTAAATGATATGCTTAAGTTCAGC		
*T. vitrinus*	**F**	**1**	ATGTGAACGTGTTGTCACTGTTTA^***§***^	150	Bisset et al. 2014
*T. axei*	**F**	**1**	GATGTTAATGTTGAACGACATTAATATC	186	
*T. colubriformis*	**R**	**1**	ACATCATACAGGAACATTAATGTCA^***#***^	232	
*T. circumcincta*	**F**	**1**	AAACTACTACAGTGTGGCTAACATA*	295	
*C. ovina*	**F**	**1**	CAGCGACTAAGAATGCTTTGG	115	
*C. curticei*	**R**	**2**	TGAGTACACTTAAACAGTGATAATAGA	264	Bisset et al. 2014
*N. spathiger*	**R**	**2**	CATTCAGGAGCTTTGACACTAAT	213	
*C. oncophora*	**R**	**2**	CTATAACGGGATTTGTCAAAACAGA	173	
*H. contortus*	**F**	**2**	CATGTATAGCGACGATGTTCTT	90	
*O. venulosum*	**R**	**2**	CGACTACGGTTGTCTCATTTCA	327	
*C. punctata*	**R**	**3**	AGATTCATATCATTCAGAAATGTTCAC	240	Master mix: 0.25 unit Platinum Taq polymerase, 1 × Taq buffer, 2.5 mM MgCl2, 200 µM each dNTP, 4 pmol of each generic primer, 3 pmol of species-specific primers Touchdown conditions as Bisset et al. 2014
*O. ostertagi*	**R**	**3**	AGTACATTCAAATAGTGGTAATATATTCAG	256	
*N. filicollis*	**R**	**3**	AATGGGATTGACTGTTACGATGTAA	165	
O. leptospicularis*°	**F**	**3**	CATGCAACATAACGTTAACATAATG	196	
*S. asymmetrica*^*§*^	**F**	**4**	GAATAACATATGCAACATAACGTTGT	210	Master mix: 0.25 unit Platinum Taq polymerase, 1 × Taq buffer, 1.5 mM MgCl2, 200 µM each dNTP, 4 pmol of each generic primer, 3 pmol of Oeve, Spas, Oera and 6 pmol of Spspi Touchdown conditions as Bisset et al. 2014
*S. spiculoptera*^*§*^	**R**	**4**	GATACATGAACAATGATTGTCATACAA	317	
*O. venulosum*	**R**	**4**	ATACATGCATGCATACATCACATG***°***	250	
*O. radiatum*^*#*^*/* *O. sikae*^*#*^	**R**	**4**	TCACAGTGACAATGAGATCACG	235	
*T. askivali*^#^	F	**5**	GTTTGTCGAATGGTCATTGTCGTAC	257	Master mix: 0.25 unit Platinum Taq polymerase, 1 × Taq buffer, 1.5 mM MgCl2, 200 µM each dNTP, 4 pmol of each generic primer, 3 pmol of Trask Touchdown conditions as Bisset et al. 2014

As the same primer was used to amplify *O. sikae* and *O. radiatum*, the PCR product from samples of all positive farms was sequenced to verify the species. In the case of *T. axei* positive samples, five ITS2 PCR products were randomly selected and sequenced. Sequences were analysed using Geneious software version 8.1 and aligned against previously described sequences of *O*. *sikae* and *O*. *radiatum* (Genbank access: KJ420898, KJ420906), or *T*. *axei* (KC998724-KC998727).

### Statistical analysis

To determine the effect of treatment on exsheathment, only those parasite species for which at least two L3 were recovered across the three replicates for each of the following treatment combinations (heat shock at pH 2, heat shock at pH 6.6, slow temperature increase at pH 2, slow temperature increase at pH 6.6) were included. Only four treatment combinations were included for the statistical analysis because no exsheathment was observed at a constant 20 °C. The dataset was further curated to exclude for each host species (1) any parasite species for which fewer than four L3 were observed across all replicates in each of the four treatments and (2) any parasite species where no exsheathment was observed (e.g. *O. venulosum* in sheep). “Pseudocounts” (one sheathed and one exsheathed observation per replicate for every species and treatment combination) were added to avoid numerical and inferential problems caused by the many conditions with zero exsheathments observed. For all analyses the significance threshold was set at *p* < 0.05. The “Results” section presents the mean exsheathment rates (%) across replicates for each species and treatment (number of exsheathed L3 for each species/number of total L3 for each species, per treatment) combination.

The treatment effect on exsheathment was then analysed by host species using a generalised linear mixed model (GLMM) with a binomial response and logistic link function. The response was one plus the number of exsheathed parasites versus one plus the number of sheathed parasites (the plus ones being the pseudocounts described above). The four treatments, farm and their interaction were included as fixed effects. For host and parasite species combinations that only used data from a single farm, only the treatment fixed effect was used. Replicate (nested in treatment nested in farm) was included as a random effect.

To investigate differences in exsheathment response between hosts, GLMM models with transformed binomial responses were used as described above. The four treatments, host and their interactions were included as fixed effects. A per-farm random effect was included for treatment, and a per-replicate random intercept was included to account for overdispersion. For *O. venulosum*, the per-farm random treatment effect was reduced to a random intercept to obtain convergence.

Analysis of the overall species composition of the samples by treatment was done by host species and was limited to farms with at least 10 L3 of that species observed at that farm across all treatments and replicates. The following treatment combinations were included: untreated control, heat shock at pH 2, heat shock at pH 6.6, slow temperature increase at pH 2, slow temperature increase at pH 6.6. Any parasite species previously excluded for exsheathment analysis was grouped as other, along with L3 which were not identified following PCR analysis. The composition analysis used GLMM with a binary response and logistic link function. Treatment and farm (where more than one was available) were included as fixed effects and replicates as random effects, and *p*-values were adjusted using the Benjamini–Hochberg correction.

## Results

### Sampling and species compositions

On average, 83 L3 (range 41–161) were present in the 1.5 mL subsamples (replicate × treatment condition) and importantly the species composition within the samples from each farm (Table 2) was not different between the experimental conditions tested (*p*-values: 0.63–0.68, 0.10–0.81 and 0.51–0.81 for cattle, deer and sheep farm species, respectively). No exsheathment occurred in any parasite species in the untreated control samples or for samples exposed to a constant 20 °C.

**Table 2:** Species composition (%) of samples obtained from sheep, cattle or deer farms. In bold: species included for exsheathment analysis

		**Farm**											
		**Sheep**				**Cattle**					**Deer**		
		**SF1**	**SF2**	**SF3**	**SF4**	**CF1**	**CF2**	**CF3**	**CF4**	**CF5**	**DF1**	**DF2**	**DF3**
**Total No. of L3 sampled for species identification**		**1293**	**1476**	**1049**	**1191**	**930**	**536**	**588**	**1460**	**953**	**757**	**704**	**934**
**Predilection site**	**Species**												
**Abomasum**	***H. contortus***	-	-	**65**	**38**	-	-	< 1	**9**	**50**	< 1	< 1	-
	***T. circumcincta***	1	**8**	**9**	**11**	-	< 1	< 1	1	**3**	1	-	< 1
	***O. ostertagi***	-	-	-	-	**30**	**29**	**29**	**6**	**17**	-	-	-
	***O. leptospicularis***	1	-	-	-	< 1	-	< 1	-	-	**42**	**3**	**16**
	***S. spiculoptera***	-	-	-	-	-	-	-	-	-	< 1	**9**	**7**
	***S. asymmetrica***	-	-	-	-	-	-	-	-	-	**22**	< 1	**10**
	***T. axei***	**15**	**10**	< 1	**4**	< 1	-	2	**45**	**20**	1	-	-
**Small intestine**	***T. colubriformis***	**33**	**60**	**11**	< 1	-	-	-	-	< 1	< 1	-	**2**
	***T. vitrinus***	**6**	**13**	**2**	**6**	< 1	-	-	< 1	**7**	< 1	-	-
	***C. curticei***	**9**	**6**	**11**	**32**	-	-	< 1	< 1	< 1	-	-	-
	***C. oncophora***	-	< 1	1	-	**69**	**71**	**67**	**34**	< 1	-	-	-
	***C. punctata***	-	-	-	-	-	-	-	-	-	-	-	-
	***N. spathiger***	-	-	-	-	-	-	-	-	< 1	-	-	-
**Large intestine**	***C. ovina***	**13**	1	-	**4**	-	-	-	-	< 1	< 1	-	-
	***O. venulosum***	19	< 1	< 1	< 1	< 1	-	-	-	1	**30**	**79**	**41**
	***O. radiatum***	-	-	-	-	-	-	-	1	-	-	-	-
	***O. sikae***	-	-	-	-	-	-	-	-	-	-	**6**	**19**
**Unidentified**		< 1	< 1	< 1	3	-	-	1	4	1	2	1	5

### Exsheathment response of parasite species from sheep

**Fig. 1:**
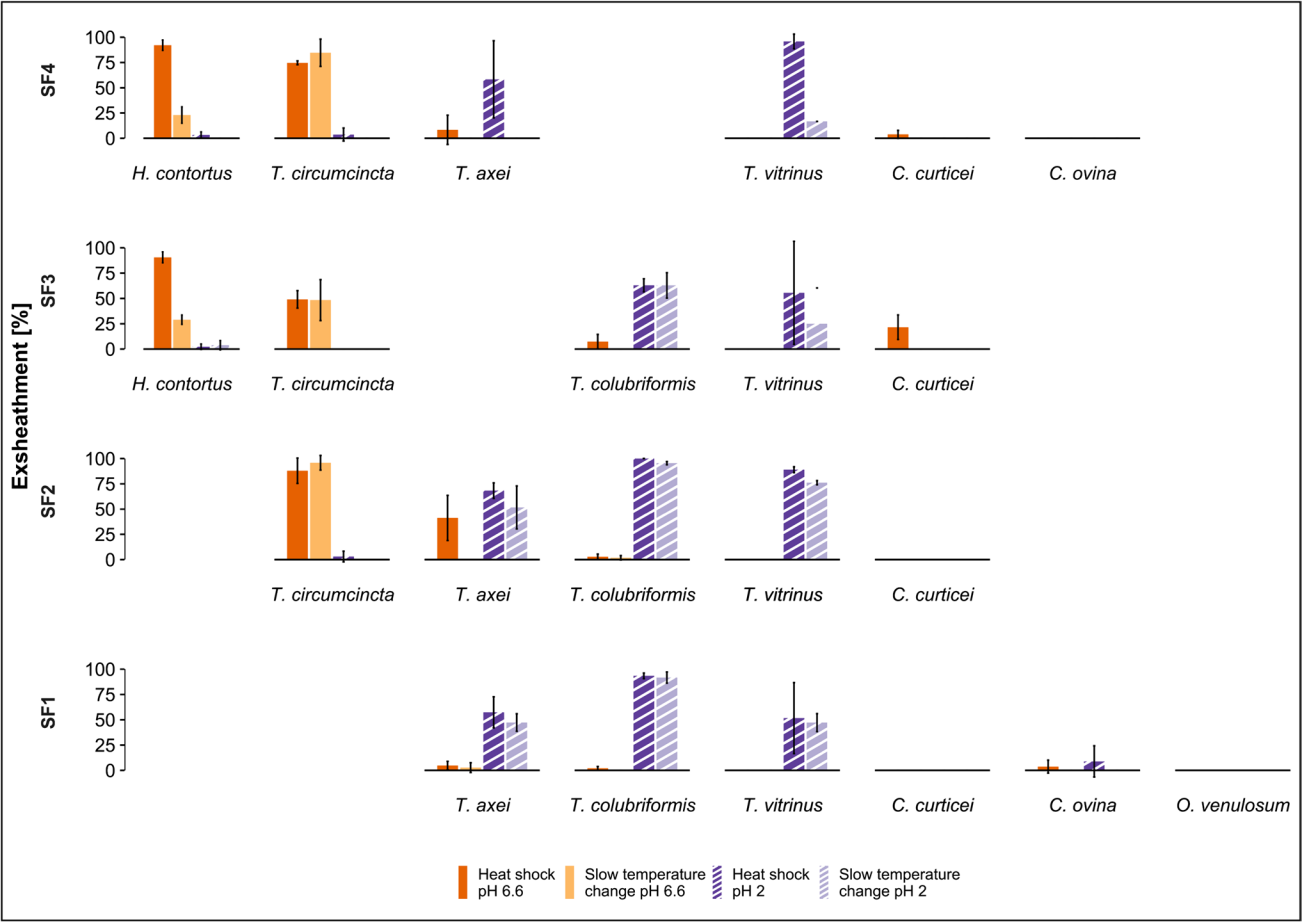
Exsheathment analysis of infective gastrointestinal nematode larvae derived from sheep: mean in vitro exsheathment rate (% exsheathed ± SEM) of each species following exposure to a heat shock (40 °C) or a slow temperature increase (from 20 to 40 °C over 4 h), at pH 2 or pH 6.6. Exsheathment is depicted for each of the four sheep farms (one farm per row). Absence of the *x*-axis and species name in a row indicates this species was not present in this sample

On sheep farms SF1 and SF2, the *Trichostrongylus* spp. were the most abundant; on SF3, *H. contortus* was dominant; and on SF4, *H. contortus* and *C. curticei* were the main species identified (Table 2). Exposure of *H. contortus* and *T. circumcincta* to heat shock and CO_2_ at pH 6.6 resulted in mean exsheathing rates of 90.2% and 67.7%, respectively, whereas exsheathment at pH 2 was significantly less (5.4% and 12.0%, respectively, *p* < 0.01) (Fig. 1, supplementary Table 1). In contrast, the *Trichostrongylus* spp. showed higher exsheathment rates when exposed to heat shock and CO_2_ at pH 2 compared to pH 6.6 (*p* < 0.01). The mean exsheathment rates at pH 2 for *T. axei*, *T. colubriformis* and *T. vitrinus* were 59.7%, 91.1% and 69.9%, respectively. Under neutral pH conditions on the other hand, only 6.8% of *T. colubriformis* and 25.7% of *T. axei* L3 exsheathed; and no exsheathment was observed for *T. vitrinus* (Fig. 1, supplementary Table 1).

An effect of the temperature component was observed for *H. contortus*, *T. vitrinus* and *T. axei*, with higher mean exsheathment rate in response to a heat shock than to a slow temperature increase. In *H. contortus* this was only seen at pH 6.6 (90.2% vs. 27.5%, *p* < 0.01); in *T. vitrinus* only at pH 2 (69.9% vs. 47.0%, *p* = 0.03); and with *T. axei*, it was observed at both pH 6.6 (25.7% vs. 11.4%, *p* = 0.04) and pH 2 (59.7% vs. 37.8%, *p* = 0.03) (Fig. 1, supplementary Table 1).

Exsheathment of *C*. *curticei* and *C. ovina* was modest (< 20%) with no significant differences observed between the different treatment conditions. No exsheathment was observed for *O. venulosum* regardless of the experimental conditions (Fig. 1, supplementary Table 1).

### Exsheathment response of parasite species from cattle

**Fig. 2:**
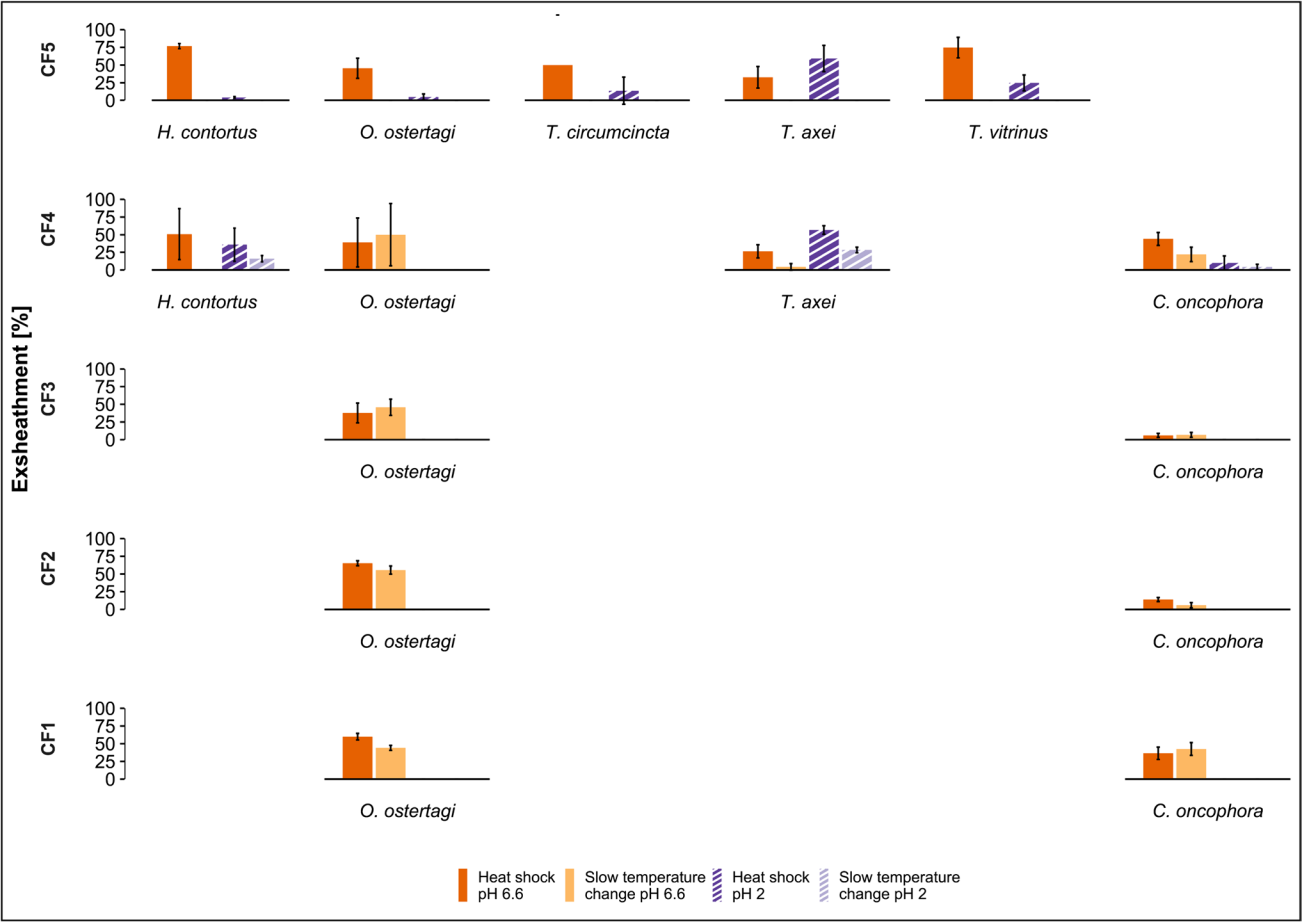
Exsheathment analysis of infective gastrointestinal nematode larvae derived from cattle: mean in vitro exsheathment rate (% exsheathed ± SEM) of each species following exposure to a heat shock (40 °C) or a slow temperature increase (from 20 to 40 °C over 4 h), at pH 2 or pH 6.6. Exsheathment is depicted for each of the five cattle farms (one farm per row). Absence of the *x*-axis and species name in a row indicates this species was not present in this sample

The most common parasites isolated from cattle were *O. ostertagi* and *C. oncophora* (Table 2) and both species showed higher exsheathment following exposure to heat shock and CO_2_ at pH 6.6 (51.6% and 22.7%, respectively) compared to pH 2 (< 10% and < 5%, respectively) (*p* < 0.01). No difference was observed between the two temperature regimes for *C. oncophora*, whereas the exsheathment rate for *O. ostertagi* was significantly higher following exposure to heat shock compared to slow temperature increase (51.6% vs 35.1%, *p* < 0.01) at pH 6.6 (Fig. 2, supplementary Table 1).

On two farms, *T. axei* was represented (Table 2) and for this parasite exsheathment was higher at pH 2 (58.5%) compared to pH 6.6 (32.4%) (*p* < 0.01). On the same farms, *H. contortus* was isolated from cattle (Table 2), and under heat shock and CO_2_, 64.7% of L3 exsheathed at pH 6.6 compared to 15.8% at pH 2 (*p* < 0.01). At pH 6.6, in both species, heat shock elicited a significantly higher response compared to slow temperature increase (*p* < 0.01). At pH 2, this result was observed only in *T. axei* (Fig. 2, supplementary Table 1)*.*

Samples from CF5 also contained *T. vitrinus* and *T. circumcincta* larvae. Both species showed an exsheathment response to heat shock but not to slow temperature increase. The mean exsheathment rate in response to heat shock and CO_2_, for *T*. *vitrinus*, was higher at pH 6.6 (73.7%) than at pH 2 (29.0%) (*p* = 0.02), whereas *T. circumcincta* exsheathed equally at both pH levels (Fig. 2, supplementary Table 1).

### Exsheathment response of parasite species from deer

**Fig. 3:**
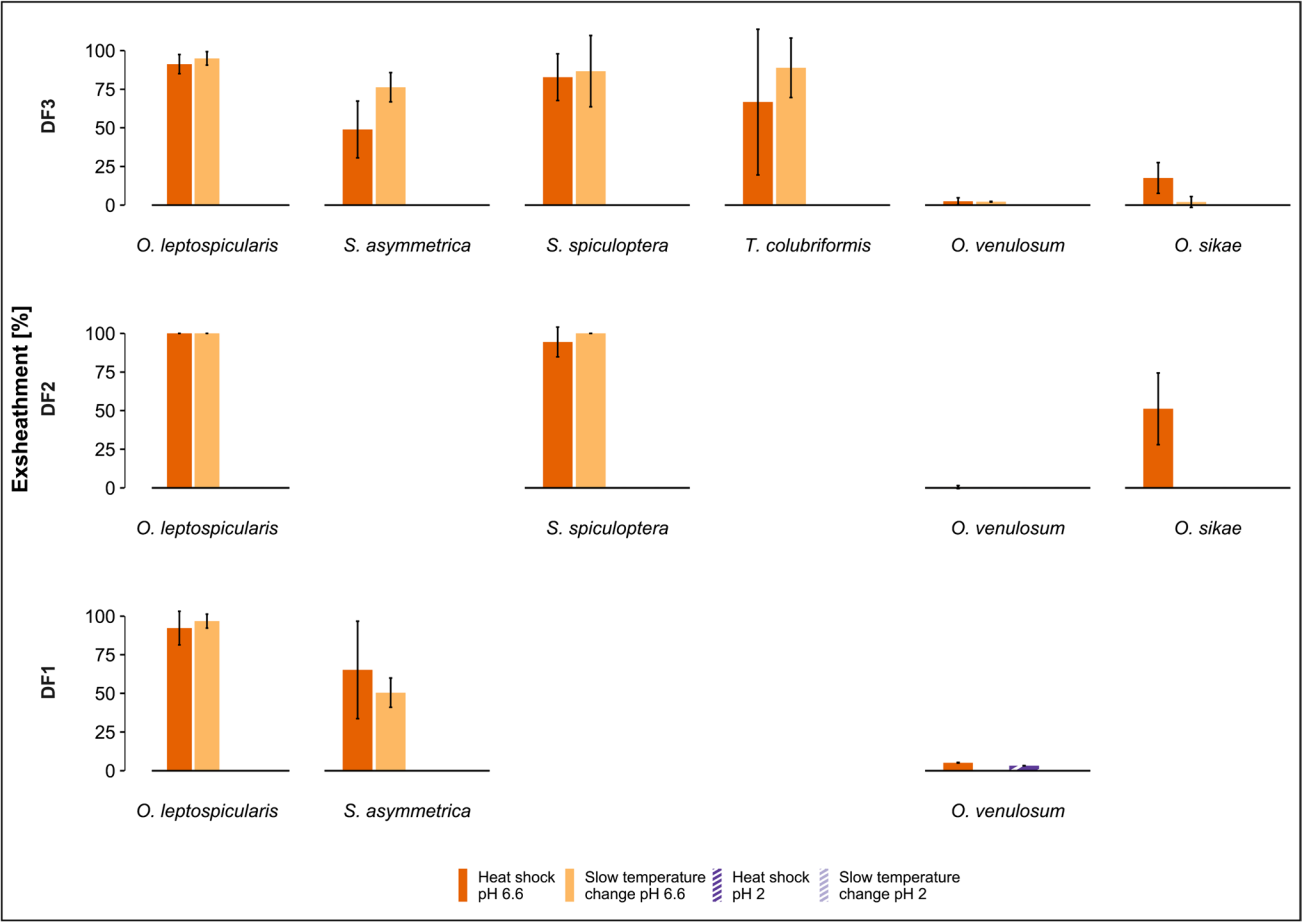
Exsheathment analysis of infective gastrointestinal nematode larvae derived from deer: mean in vitro exsheathment rate (% exsheathed ± SEM) of each species following exposure to a heat shock (40 °C) or a slow temperature increase (from 20 to 40 °C over 4 h), at pH 2 or pH 6.6. Exsheathment is depicted for each of the three deer farms (one farm per row). Absence of the *x*-axis and species name in a row indicates this species was not present in this sample

The most prevalent parasites recovered from deer were *Oesophagostomum* spp. and the Ostertagiinae complex (Table 2). The three Ostertagiinae spp. (*O. leptospicularis*, *S. asymmetrica* and *S. spiculoptera*) showed high levels of exsheathment (85.7%, 53.9% and 78.2%, respectively), which was only achieved at pH 6.6 (*p* < 0.01), and there were no significant differences between the two temperature regimes (Fig. 3, supplementary Table 1). Exsheathment of *O. sikae* was also observed exclusively at pH 6.6, and was significantly higher following exposure to heat shock compared to slow temperature increase (32.9% vs 9.2%, *p* = 0.03). In contrast, the exsheathment rate of *O. venulosum* was < 5% under all the test conditions (Fig. 3, supplementary Table 1). In samples from DF3, a small number of *T. colubriformis* L3 were found and they showed exsheathment only at pH 6.6, with no significant differences between the two temperature regimes (Fig. 3, supplementary Table 1).

### Parasite exsheathment response across host species

The parasite species *H. contortus*, *T. axei*, *T. vitrinus* and *T. circumcincta* were present in samples derived from cattle and sheep, and *T. colubriformis* was recovered from sheep as well as deer samples. With the exception of *T. axei*, the host species affected the response to the different exsheathment conditions. For *H. contortus*, *T. vitrinus* and *T. circumcincta*, the effect of the host was observed only at pH 6.6. Exsheathment at both temperature regimes was higher in *H. contortus* from sheep than from cattle (*p* < 0.01). Unlike sheep, no exsheathment in response to slow temperature increase was measured in either *H. contortus* or *T*. *circumcincta* from cattle. In the case of *T. vitrinus*, exsheathment in response to heat shock was observed only in parasites from cattle. *T. colubriformis* parasites from deer had higher exsheathment at pH 6.6 in both temperature regimes, while at pH 2, exsheathment was only observed in parasites from sheep.

## Discussion

The primary aim of this study was to look for commonality in exsheathment triggers across a range of nematode species from different host species. There was an a priori expectation that species which reside in the same organ within the gastrointestinal track would exhibit similarity in triggers, especially with respect to pH. Previous in vivo works indicated that exsheathment occurs mainly, but not exclusively, in the organ preceding the predilection site (Sommerville 1954, 1957). Hence, for species that establish in the abomasum, exsheathment would typically occur in the rumen, in an environment with a relatively neutral pH. Exsheathment of *H. contortus*, *T. circumcincta*, *O. ostertagi* and *O. leptospicularis* has previously been triggered successfully at a neutral pH (Bekelaar et al. 2018a, 2019), and the optimal pH for in vitro exsheathment of *H. contortus* has been shown to be between 6 and 8 (Rogers 1960; Silverman and Podger 1964). In the current study, responses were measured to heat acclimation, CO_2_ and pH, because for some nematode species these factors alone trigger > 90% exsheathment in vitro (Bekelaar et al. 2018a, 2019), indicating that they alone can meet the requirements for exsheathment in vivo.

The initial expectation of similarity in pH requirements amongst species residing within the same organ was not supported. All the members of the subfamily Ostertagaiinae and *H*. *contortus* showed a strong preference for a neutral pH, regardless of host species, which might be expected given they exsheath in the rumen and reside in the abomasum. In contrast, *T*. *axei*, also an abomasal species, showed a preference for the lower pH regardless of whether the larvae were isolated from sheep or cattle. Previously, Rogers (1960) found high levels of exsheathment of this species at pH 6–8, but did not measure exsheathment at lower pHs. A full sequence analysis of the ITS2 region for individual *T. axei* L3 exsheathed at pH 2 confirmed the identity of the species (99.5% identical to *T. axei* sequences retrieved from Genbank). Due to differences in the experimental setup (e.g. the in vitro medium and exposure times), a direct comparison between both studies is not feasible. The in vivo study by Sommerville (1957) appears to support the hypothesis that *T. axei* might be capable of exsheathment in both the rumen and abomasum. However, some caution is warranted when interpreting these data as the study endpoint was not exsheathment itself but rather the emergence of the refractile ring, i.e. completion of the first phase in the exsheathment process. Refractile ring formation can be stimulated by sub-optimal conditions and is not always followed by a completion of the exsheathment process (Smales and Sommerville 1977). It may be significant for interpreting these findings, that *T*. *axei* is a generalist parasite, capable of establishing viable infections in a range of host species, including sheep, goats, cattle, deer and horses (Sutherland and Scott 2010). A wide host range may coincide with an ability to exsheath under a wider range of environmental triggers.

Although they did show some ambiguity in pH requirements depending on their host, both *T. vitrinus* and *T*. *colubriformis* showed a preference for pH 2 when isolated from sheep, consistent with a predilection site in the small intestine. For the latter species, this was in line with previous reports (Hertzberg et al. 2002; Rogers 1960; Silverman and Podger 1964; Sommerville 1957). In contrast to these intestinal *Trichostrongylus* spp., the intestinal *C*. *oncophora* showed a preference for pH 6.6, while *C*. *curticei* showed no preference for either pH exhibiting a relatively poor exsheathment (< 20%) in both.

These inconsistencies in pH preference, or lack thereof, are difficult to explain. Some flexibility in pH requirements for species such as *T*. *axei*, might be expected given its ability to establish and thrive in a range of host species. However, another abomasal species with the same multi-host capacity (i.e. *O*. *leptospicularis*) showed a clear preference for neutral pH. Given that these are both abomasal parasites with a broad host range, this explanation is not compelling. The *Cooperia* spp. are generally considered small intestinal parasites and would therefore be expected to exsheath in the abomasum. However, exsheathment of *C. oncophora* was observed predominantly at pH 6.6. Previous reports describing *Cooperia* exsheathment in rumen fluid proposed that these may originally have been abomasal species that have subsequently adapted to the small intestine, a suggestion based on the prevalence of *Cooperia* in the abomasum for some hosts (Ahluwalia and Charleston 1974; Chollet et al. 2000; Hertzberg et al. 2002). This could help explain current findings around pH preferences for these species.

The large intestinal nematodes *C. ovina* and *O. venulosum* recovered from sheep and deer showed no, or extremely limited, exsheathment in response to CO_2_ and heat shock regardless of pH, suggesting that these species rely on entirely different exsheathment triggers. This is supported by the lack of exsheathment of these species in rumen fluid at both neutral and low pH (Hertzberg et al. 2002). Potential trigger candidates are a change from low to neutral pH, metabolic components, or the different gall bladder and pancreatic enzymes present in the small intestine (Joachim et al. 2005). However, the deer parasite *O. sikae* appeared more responsive to heat shock and CO_2_ compared to the other two large intestinal species: an average exsheathment of 32.9% was observed at pH 6.6, but no exsheathment occurred at pH 2. It would be interesting to identify the exsheathment location of *O. sikae* compared to *C. ovina* and *O. venulosum*, which are thought to exsheath in the small intestine (Hertzberg et al. 2002; Sommerville 1957).

Previous studies have shown that the temperature requirements for exsheathment of *H*. *contortus* are dependent on the rate and magnitude of the temperature change (Bekelaar et al. 2018a). As was the case with pH, in this study, there were differences between parasite species in their response to temperature acclimation. Five species had a higher mean exsheathment in response to heat shock, i.e. the abomasal species *H. contortus*, *O. ostertagi* and *T. axei*, the small intestinal *T. vitrinus*, and the large intestinal *O. sikae*. But these species also showed differences in their preference for heat shock combined with pH, with *H. contortus* and *O. ostertagi* only showing a preference for heat shock at pH 6.6, while *T*. *vitrinus* preferred heat shock at pH 2 and *T. axei* reacted similarly at both pHs. Other species showed no preference for heat shock. Bekelaar et al. (2018a) speculated that heat shock might be important as an exsheathment trigger because if L3 take 3–4 days to exsheath (in response to just elevated temperature), then by the time they have exsheathed, they would have passed so far down the alimentary tract that they would have missed their predilection sites and be unable to establish. It may be that the lack of response to heat shock is due to the absence of additional triggers, which appear necessary for some species to achieve high levels of exsheathment (Bekelaar et al. 2019).

The host in which a parasite establishes has been shown to influence a range of epidemiological parameters in the progeny of those worms after they leave the host in the free-living stages (Sauermann et al. 2021; Siamba et al. 2012). Also, although some parasite species can complete their life cycle in non-preferred hosts, their ability to do so is often reduced (Waghorn et al. 2024). It is perhaps not surprising, then, that in this study sheep species isolated from cattle tended to show a reduced exsheathment percentage and a narrower range of trigger conditions. There were, however, several unexplained observations with *T*. *vitrinus* showing a preference for pH 6.6, and responding to heat shock when isolated from cattle but not when isolated from sheep. Similarly, *T*. *colubriformis* from deer showed a preference for pH 6.6 while those from sheep preferred pH 2.

It must be noted that *T. vitrinus* and *T. colubriformis* were only found in sufficient numbers for inclusion in one of the cattle- or deer-derived samples, respectively. As a result, it is unclear whether these discrepancies in their exsheathment triggers when compared to sheep-derived samples are an unusual outlier, or whether it is in fact driven by the different host species. A number of species (*T. circumcincta*, *T. colubriformis* and *T. vitrinus*) demonstrated a capacity for a high exsheathment response to the tested triggers, but also showed variability between cohorts (> 50% difference in exsheathment percentage across farms). Variability in exsheathment response between different cohorts of larvae has been described previously (Petronijevic et al. 1985, 1986; Rogers 1960; Sauermann et al. 2021), although it remains unclear what drives these differences. A possible explanation is that the L3 fitness, and exsheathment potential, is related to the larva’s prior exposure to environmental factors (e.g. low/high temperatures, desiccation, etc.) or to intergenerational effects from its progenitor’s individual host (e.g. host age or immunogenic potential) (Sauermann et al. 2021; Siamba et al. 2012). Hence, the variability in response observed from single samples of *T. vitrinus* and *T. colubriformis* from alternate hosts must be viewed with caution.

In the presence of these in vitro triggers, exsheathment of some species was high (> 90%), whereas for other species exsheathment percentage was consistently low under all test conditions (e.g. < 20% for *C*. *ovina* and *C*. *curticei* and < 5% for *O*. *venulosum*). These low values suggest that other factors are likely required to achieve full exsheathment potential. This has been demonstrated previously, where *H*. *contortus* and *O*. *leptospicularis* exsheathed equally in rumen fluid and artificial buffer, but for *O*. *ostertagi* and *T*. *circumcincta* exsheathment was significantly lower in buffer compared to rumen fluid (Bekelaar et al. 2019), suggesting that additional factors present in the rumen fluid were required. The lower exsheathment response of the *Cooperia* spp. reported here, compared to previous studies in rumen fluid (Ahluwalia and Charleston 1974; Hertzberg et al. 2002), further supports this hypothesis.

The current method of using convenient sampling of farms allowed for a more realistic insight into the exsheathment response of larvae being deposited onto the farm. These are natural populations, and they were tested in naturally occurring combinations, as would happen in the grazing host animal. An additional benefit is that this approach overcomes the limitations associated with the need to obtain pure GIN cultures. This allowed for the inclusion of a wider range of species as well as larvae derived from a non-preferred host for a number of species, resulting in some surprising findings. The potential drawback of using a mixture of species is that one species exsheathing might stimulate exsheathment of other species as well when larvae are concentrated enough. Evidence for such an effect is rather limited as it looks at refractile ring formation (which does not necessarily translate into exsheathment (Smales and Sommerville 1977; Sommerville 1957; Sommerville and Murphy 1983), and uses either concentrated exsheathment fluid or high concentrations of larvae (1000 to 10,000 L3 per mL compared to 60 per mL in the current study) (Rogers and Sommerville 1960; Silverman and Podger 1964; Sommerville 1957). Nevertheless, the number and concentration of the larvae in the exsheathment assay was kept to a minimum to mitigate this potential issue. Given the obvious differences between species, it is unlikely that exsheathment of one species induced a response in the others.

The data show a perhaps surprising variability between nematode species in their response to these in vitro exsheathment triggers, and inconsistency between species residing in the same organ within the intestinal tract. Given the complexity of responses shown by different nematode species to this range of exsheathment triggers, it will likely be difficult to find a single trigger, or set of triggers, that will stimulate all species to exsheath. Equally, finding a single compound capable of blocking exsheathment in all species appears unlikely. However, for some parasite species where these triggers work effectively, their use may find application in in vitro testing of compounds for anthelmintic efficacy, hence negating the more artificial use of caustic compounds to exsheath larvae. Perhaps the best option is to identify key components of the exsheathment process following its initiation, common to all species, in the hope that this can be targeted as a new means of preventing parasite infection.

### Supplementary Information

Below is the link to the electronic supplementary material.

Supplementary file1 (DOCX 34 kb)

## Data Availability

All data supporting the findings of this study are available within the paper.
