# Supplementary material for: Inconsistency of in vitro exsheathment triggers for gastrointestinal nematode parasites of sheep, cattle and deer
Source: Parasitol Res. 2024 Jul 11;123(7):267. doi: 10.1007/s00436-024-08277-z (PMC11239767; doi:10.1007/s00436-024-08277-z)
Supplement: Supplementary file 1 — Supplementary file1 (DOCX 34 kb) [file 436_2024_8277_MOESM1_ESM.docx]

**Supplementary table 1**: Table complementary to Figures 1-3. Mean exsheathment rate per species and per host under the different treatment conditions. Comparison of mean exsheathment rates (%) and p values for each evaluated species between the different treatment conditions. CI: confidence interval, HS: heat shock (40°C), SU: slow temperature change (from 20°C to 40°C over 4 h).

|  |  |  |  |  | **Comparison p-value** | | |
| --- | --- | --- | --- | --- | --- | --- | --- |
|  | **Species** | **Treatment** | **Percentage** | **CI** | **SU pH6** | **HS pH2** | **SU pH2** |
|  |  |  |  |  |  |  |  |
| **CATTLE** | *Cooperia oncophora* | HS pH6 | 22.7 | 19.0 - 26.9 | 0.08 | <0.01 | <0.01 |
|  |  | SU pH6 | 17.4 | 13.9 - 21.6 |  | <0.01 | <0.01 |
|  |  | HS pH2 | 3.5 | 2.1 - 5.9 |  |  | 0.63 |
|  |  | SU pH2 | 2.9 | 1.7 - 5.0 |  |  |  |
|  | *Ostertagia ostertagi* | HS pH6 | 51.6 | 44.4 - 58.8 | <0.01 | <0.01 | <0.01 |
|  |  | SU pH6 | 35.1 | 27.8 - 43.3 |  | <0.01 | <0.01 |
|  |  | HS pH2 | 6.8 | 4.0 - 11.1 |  |  | 0.73 |
|  |  | SU pH2 | No exsheathment observed |  |  |  |  |
|  | *Trichostrongylus axei* | HS pH6 | 32.4 | 26.1 - 39.4 | <0.01 | <0.01 | 0.03 |
|  |  | SU pH6 | 7.2 | 3.5 - 13.9 |  | <0.01 | 0.06 |
|  |  | HS pH2 | 58.5 | 50.3 - 66.2 |  |  | <0.01 |
|  |  | SU pH2 | 16.4 | 9.1 - 27.7 |  |  |  |
|  | *Haemonchus contortus* | HS pH6 | 64.7 | 55.5 - 72.9 | <0.01 | <0.01 | <0.01 |
|  |  | SU pH6 | No exsheathment observed |  |  | 0.09 | 0.64 |
|  |  | HS pH2 | 15.8 | 10.4 - 23.4 |  |  | 0.11 |
|  |  | SU pH2 | 7.8 | 3.7 - 15.8 |  |  |  |
|  | *Trichostrongylus vitrinus* | HS pH6 | 73.7 | 54.0 - 87.0 | 0.02 | 0.02 | 0.02 |
|  |  | SU pH6 | No exsheathment observed |  |  | 0.44 | 1 |
|  |  | HS pH2 | 29 | 13.8 - 51.0 |  |  | 0.44 |
|  |  | SU pH2 | No exsheathment observed |  |  |  |  |
|  | *Teladorsagia circumcinta* | HS pH6 | 50 | 14.0 - 86.0 | 0.55 | 0.55 | 0.55 |
|  |  | SU pH6 | No exsheathment observed |  |  | 0.55 | 1 |
|  |  | HS pH2 | 31.2 | 10.2 - 64.5 |  |  | 0.55 |
|  |  | SU pH2 | No exsheathment observed |  |  |  |  |

|  |  |  |  |  | **Comparison p-value** | | |
| --- | --- | --- | --- | --- | --- | --- | --- |
|  | **Species** | **Treatment** | **Percentage** | **CI** | **SU pH6** | **HS pH2** | **SU pH2** |
|  |  |  |  |  |  |  |  |
| **DEER** | *Oesophagostomum venulosum* | HS pH6 | 4.6 | 2.6 - 8.0 | 0.77 | 0.77 | 0.77 |
|  |  | SU pH6 | 2.5 | 1.3 - 4.9 |  | 0.77 | 0.77 |
|  |  | HS pH2 | 3.7 | 1.8 - 7.8 |  |  | 0.77 |
|  |  | SU pH2 | No exsheathment observed |  |  |  |  |
|  | *Ostertagia leptospicularis* | HS pH6 | 85.7 | 76.3 - 91.7 | 0.72 | <0.01 | <0.01 |
|  |  | SU pH6 | 88.2 | 79.8 - 93.4 |  | <0.01 | <0.01 |
|  |  | HS pH2 | No exsheathment observed |  |  |  | 0.88 |
|  |  | SU pH2 | No exsheathment observed | 0.0 - 18.9 |  |  |  |
|  | *Oesophagostomum sikae* | HS pH6 | 32.9 | 21.1 - 47.5 | 0.03 | 0.11 | 0.11 |
|  |  | SU pH6 | 9.2 | 4.4 - 18.3 |  | 0.62 | 0.62 |
|  |  | HS pH2 | No exsheathment observed |  |  |  | 0.88 |
|  |  | SU pH2 | No exsheathment observed |  |  |  |  |
|  | *Spiculopteragia asymmetrica* | HS pH6 | 53.9 | 39.0 - 68.1 | 0.44 | <0.01 | <0.01 |
|  |  | SU pH6 | 62 | 48.9 - 73.6 |  | <0.01 | <0.01 |
|  |  | HS pH2 | No exsheathment observed |  |  |  | 0.67 |
|  |  | SU pH2 | No exsheathment observed |  |  |  |  |
|  | *Spiculopteragia spiculoptera* | HS pH6 | 78.2 | 63.3 - 88.2 | 0.54 | <0.01 | <0.01 |
|  |  | SU pH6 | 84.4 | 70.0 - 92.7 |  | <0.01 | <0.01 |
|  |  | HS pH2 | No exsheathment observed |  |  |  | 0.97 |
|  |  | SU pH2 | No exsheathment observed |  |  |  |  |
|  | *Trichostrongylus colubriformis* | HS pH6 | 60 | 22.2 - 88.7 | 1 | 0.31 | 0.31 |
|  |  | SU pH6 | 63.6 | 25.9 - 89.8 |  | 0.31 | 0.31 |
|  |  | HS pH2 | No exsheathment observed |  |  |  | 1 |
|  |  | SU pH2 | No exsheathment observed |  |  |  |  |

|  |  |  |  |  | **Comparison p-value** | | |
| --- | --- | --- | --- | --- | --- | --- | --- |
|  | **Species** | **Treatment** | **Percentage** | **CI** | **SU pH6** | **HS pH2** | **SU pH2** |
|  |  |  |  |  |  |  |  |
| **SHEEP** | *Trichostrongylus colubriformis* | HS pH6 | 6.8 | 4.2 - 10.7 | 0.52 | <0.01 | <0.01 |
|  |  | SU pH6 | 5.3 | 2.8 - 9.7 |  | <0.01 | <0.01 |
|  |  | HS pH2 | 91.1 | 85.9 - 94.5 |  |  | 0.12 |
|  |  | SU pH2 | 85.4 | 79.2 - 89.9 |  |  |  |
|  | *Haemonchus contortus* | HS pH6 | 90.2 | 85.5 - 93.5 | <0.01 | <0.01 | <0.01 |
|  |  | SU pH6 | 27.5 | 22.0 - 33.7 |  | <0.01 | <0.01 |
|  |  | HS pH2 | 5.4 | 2.8 - 9.9 |  |  | 0.68 |
|  |  | SU pH2 | 4.5 | 2.2 - 8.9 |  |  |  |
|  | *Cooperia curticei* | HS pH6 | 11.6 | 7.2 - 18.0 | 0.78 | 0.78 | 0.78 |
|  |  | SU pH6 | No exsheathment observed |  |  | 0.78 | 0.78 |
|  |  | HS pH2 | No exsheathment observed |  |  |  | 0.78 |
|  |  | SU pH2 | No exsheathment observed |  |  |  |  |
|  | *Trichostrongylus axei* | HS pH6 | 25.7 | 16.6 - 37.6 | 0.04 | <0.01 | 0.15 |
|  |  | SU pH6 | 11.4 | 6.0 - 20.7 |  | <0.01 | <0.01 |
|  |  | HS pH2 | 59.7 | 48.3 - 70.2 |  |  | 0.03 |
|  |  | SU pH2 | 37.8 | 26.1 - 51.2 |  |  |  |
|  | *Trichostrongylus vitrinus* | HS pH6 | No exsheathment observed |  | 0.95 | <0.01 | <0.01 |
|  |  | SU pH6 | No exsheathment observed |  |  | <0.01 | <0.01 |
|  |  | HS pH2 | 69.9 | 58.4 - 79.4 |  |  | 0.03 |
|  |  | SU pH2 | 47 | 32.0 - 62.5 |  |  |  |
|  | *Teladorsagia circumcinta* | HS pH6 | 67.7 | 57.6 - 76.3 | 0.72 | <0.01 | <0.01 |
|  |  | SU pH6 | 71.5 | 58.8 - 81.5 |  | <0.01 | <0.01 |
|  |  | HS pH2 | 12 | 6.5 - 21.1 |  |  | 0.84 |
|  |  | SU pH2 | No exsheathment observed |  |  |  |  |
|  | *Chabertia ovina* | HS pH6 | 14.8 | 6.7 - 29.8 | 0.8 | 0.81 | 0.93 |
|  |  | SU pH6 | No exsheathment observed |  |  | 0.8 | 0.8 |
|  |  | HS pH2 | 19.3 | 9.3 - 35.6 |  |  | 0.81 |
|  |  | SU pH2 | No exsheathment observed |  |  |  |  |
